# Supplementary material for: Application of “Magnetic Anchors” to Align Collagen Fibres for Axonal Guidance
Source: Gels. 2021 Sep 27;7(4):154. doi: 10.3390/gels7040154 (PMC8544430; doi:10.3390/gels7040154)
Supplement: Supplementary file 1 [file gels-07-00154-s001.zip › gels-1348765-supplementary.pdf]

Electronic Supplementary Information

Application of “Magnetic Anchors” to Align Collagen Fibres for Axonal Guidance

Devindraan S/O Sirkkunan, Farina Muhamad * and Belinda Pingguan-Murphy

Department of Biomedical Engineering, Faculty of Engineering, Universiti Malaya, Kuala Lumpur 50603,
Malaysia; ezvn2066@gmail.com (D.S.); bpingguan@gmail.com (B.P.-M.)
* Correspondence: farinamuhamad@um.edu.my

Table S1. The different groups of collagen scaffold fabrication methods compared and their corresponding constituents summarized in this table.

Scaffold Types	Constituents of Complete Collagen Solution			Final Fabrication Procedure
Plain Collagen	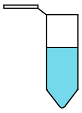 Collagen (833 μl) + 10× PBS (100 μl) + NaOH (21 μl)	dH_2O (46 μl)/~46,000 PC12 Cells in RPMI1640 culture medium (46 μl)		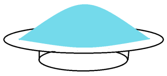 Complete collagen solution pipetted on mould before solidification into collagen scaffolds
GMNP incorporated collagen scaffolds without magnetic field treatment	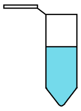 Collagen (833 μl) + 10× PBS (100 μl) + NaOH (21 μl)	dH_2O (46 μl)/~46,000 PC12 Cells in RPMI1640 culture medium (46 μl)	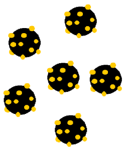 GMNP (0.506 mg)	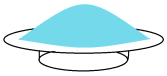 Complete collagen solution pipetted on mould before solidification into collagen scaffolds
Scaffolds fabricated with the "magnetic particle string" method	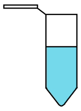 Collagen (833 μl) + 10× PBS (100 μl) + NaOH (21 μl)	dH_2O (46 μl)/~46,000 PC12 Cells in RPMI1640 culture medium (46 μl)	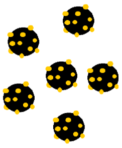 GMNP (0.506 mg)	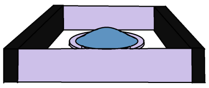 Complete collagen solution was pipetted on moulds within a magnetic field set up before solidification into collagen scaffolds
CMP functionalized GMNP incorporated collagen scaffolds without magnetic field treatment	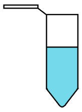 Collagen (833 μl) + 10× PBS (100 μl) + NaOH (21 μl)	dH_2O (46 μl)/~46,000 PC12 Cells in RPMI1640 culture medium (46 μl)	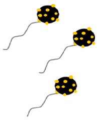 GMNPs (0.505 mg) functionalized with CMPs (0.0575 mg)	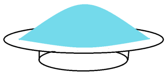 Complete collagen solution pipetted on mould before solidification into collagen scaffolds
Scaffolds fabricated with the "magnetic anchor" method	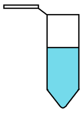 Collagen (833 μl) + 10× PBS (100 μl) + NaOH (21 μl)	dH_2O (46 μl)/~46,000 PC12 Cells in RPMI1640 culture medium (46 μl)	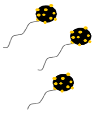 GMNPs (0.505 mg) functionalized with CMPs (0.0575 mg)	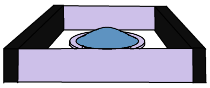 Complete collagen solution was pipetted on moulds within a magnetic field set up before solidification into collagen scaffolds

Table S2. The angle of deviation from the mean of all the PC12 neurites of the different groups of scaffolds compared in this study. 25 cells were chosen from each scaffold and their neurite's angle of deviation from the mean is shown here.

Scaffolds faricated with the "magnetic particle string" method angle of deviation from mean (°)	CMP functionalized GMNP scaffolds without magnetic field treatment angle of deviation from mean (°)	Scaffolds Fabricated with the "magnetic anchor" method angle of deviation from mean (°)
6.943	0.01222	17.1686
10.147	0.82778	9.1114
20.583	3.17222	13.3486
7.697	4.05778	5.2814
43.957	12.30778	16.9814
75.983	15.33222	22.2886
65.303	24.79778	111.6814
19.863	27.16222	21.6386
72.787	27.20222	10.7886
60.213	34.13778	2.1086
44.933	35.55222	18.8386
95.987	36.6878	19.3086
95.987	39.64222	17.2086
73.353	40.40778	13.6486
63.117	40.45222	51.2186
51.857	41.03222	9.0486
76.617	42.71778	13.0286
48.423	43.78222	50.1186
42.737	44.36778	25.2814
9.723	49.90222	33.6286
33.823	50.52778	10.1486
19.307	50.81778	17.1586
8.363	52.60222	5.0914
1.513	52.80778	16.7714
17.293	53.22778	7.6214
80.676	54.50222	2.5986
2.617	57.55778	16.4486
14.417	58.49778	26.1514
64.157	61.32222	0.6486
49.903	61.65222	22.4814
58.303	63.90778	19.0314
67.617	67.58222	6.1014
24.973	67.75222	18.1586
54.863	68.40222	32.8214
77.323	68.73222	4.7086
86.327	69.04222	19.1314
23.437	70.06222	3.4886

46.593	70.08778	16.9814
73.973	71.26222	21.1914
73.343	71.91778	15.1514
84.487	72.22222	6.6414
58.117	73.29778	32.2014
59.317	73.34778	28.8386
10.873	73.36222	0.1186
7.497	74.43222	
60.813	76.01222	
24.653	77.37778	
10.557	79.63222	
19.703	83.58778	
53.527	84.18778	
	85.40778	
	89.43778	
	94.63778	
	97.76778	

Table S3. The number of neurites and its average angle of deviation from the mean for the different group of scaffolds compared in this study. The number of neurites and angle of deviation from the mean represented in this table is an average from the 25 cells chosen from each scaffold types.

	Scaffolds fabricated with the "magnetic particle string" method	CMP functionalized GMNP collagen scaffolds without magnetic field treatment	Scaffolds fabricated with the "magnetic anchor" method
Number on neurites per 25 cells	49	53	43
Average angle of deviation from mean (°)	45.0915	54.45426	18.89572

Table S4. The angle and probability density function of every neurites from the scaffolds compared this study.

Scaffolds faricated with the "magnetic particle string" method		CMP functionalized GMNP scaffolds without magnetic field treatment		Scaffolds Fabricated with the "magnetic anchor" method	
Angle (°)	Probability Density Function	Angle (°)	Probability Density Function	Angle (°)	Probability Density Function
6.69	0.002552	1.68	0.001746	17.1	1.68E-06
8.03	0.00265	4.81	0.0019	95.96	0.006942
10.04	0.002801	10.01	0.002173	96.58	0.007149
10.66	0.002848	14.04	0.0024	102.63	0.009253
10.67	0.002849	15.26	0.00247	103.5	0.00956
18.71	0.003495	15.86	0.002506	106.3	0.010541
23.2	0.003878	22.07	0.002883	107.59	0.010984
23.8	0.00393	26.1	0.00314	109.65	0.011671
25.71	0.004096	26.15	0.003144	109.75	0.011704
29.15	0.004399	27.53	0.003233	111.8	0.012353
33.37	0.004774	29.36	0.003354	111.8	0.012353
34.11	0.004839	35.54	0.003767	112.01	0.012417
35.59	0.00497	41.89	0.004199	113.63	0.012895
37.42	0.005131	46.22	0.004491	119.67	0.014352
39.08	0.005276	46.64	0.00452	121.16	0.014616
50.19	0.006194	48.63	0.004653	122.14	0.014766
59.04	0.006815	48.92	0.004672	122.68	0.01484
59.36	0.006835	55.08	0.005072	123.5	0.014942
63.43	0.007069	56.73	0.005175	123.69	0.014963
64.15	0.007107	59.04	0.005317	128.9	0.015249
64.31	0.007115	62.76	0.005535	129.43	0.015245
66.72	0.007233	65.31	0.005678	130.89	0.0152
73.14	0.007477	74.65	0.006134	131.38	0.015174
74.29	0.007509	87.14	0.006547	132.27	0.015114
75.65	0.007543	95.39	0.006672	133.49	0.015004
77.07	0.007573	98.62	0.006687	137.83	0.014364
82.5	0.007637	99.46	0.006688	138.93	0.014144
86.63	0.007631	102.62	0.006678	139.57	0.014006
91.51	0.007562	114.78	0.00647	141.81	0.013471
91.71	0.007558	126.55	0.006032	142.13	0.013388
94.16	0.007498	126.61	0.006029	142.43	0.013309
94.57	0.007486	135	0.0056	145.23	0.012514
98.43	0.007355	139.09	0.005314	145.94	0.012298
103.32	0.007136	139.9	0.005314	145.95	0.012295
107.45	0.006908	140.48	0.005279	145.99	0.012283
126.75	0.005466	143.23	0.005109	146.94	0.011985
127.97	0.005361	149.35	0.004713	147.62	0.011767

135.87	0.004666		152.05	0.004533		148.09	0.011614
137.54	0.004518		153.95	0.004406		150.42	0.010832
142.13	0.004112		157.94	0.004135		151.07	0.010608
143.33	0.004008		160.77	0.003943		157.62	0.008306
147.13	0.00368		161.1	0.00392		162.41	0.006675
148.17	0.003592		167.03	0.00352		178.9	0.002434
151.63	0.003303		167.2	0.003509		180	0.002243
156.8	0.002892		167.85	0.003465			
160.63	0.002604		168.18	0.003443			
168.5	0.002063		168.49	0.003423			
170.34	0.001948		169.51	0.003355			
180	0.00141		170.71	0.003276			
180	0.00141		171.67	0.003214			
			172.81	0.003139			
			173.88	0.00307			
			175.46	0.00297			
			179.08	0.002744			

Table S5. The analysis of the data for angle and probability density function of the PC12 neurites from the different scaffolds compared for ANOVA.

Treatment →	Scaffolds faricated with the "magnetic particle string" method	CMP functionalized GMNP scaffolds without magnetic field treatment	Scaffolds Fabricated with the "magnetic anchor" method	Pooled Total
observations N	50	54	44	148
sum ∑xi∑xi	2254.58	2940.53	831.4116	6026.52
mean ¯xx¯	45.0915	54.4543	18.8957	40.7197
sum of squares ∑x2i∑xi2	140,262.40	192,161.00	30,114.03	362,537.44
sample variance s2s2	787.7599	604.4644	334.9748	796.867
sample std. dev. ss	28.0671	24.5859	18.3023	28.2288
std. dev. of mean SE¯xSEx¯	3.9693	3.3457	2.7592	2.3204

Table S6. The Tukey post hoc test of the ANOVA test performed for data for angle and probability density function of the PC12 neurites.

treatments	Tukey HSD	Tukey HSD	Tukey HSD
pair	Q statistic	p-value	inferfence
Scaffolds faricated with the "magnetic particle string" method vs CMP functionalized GMNP scaffolds without magnetic field treatment	2.7858	0.1235283	insignificant
Scaffolds faricated with the "magnetic particle string" method vs Scaffolds Fabricated with the "magnetic anchor" method	7.4006	0.0010053	** p<0.01
CMP functionalized GMNP scaffolds without magnetic field treatment vs Scaffolds Fabricated with the "magnetic anchor" method	10.2244	0.0010053	** p<0.01

Table S7. The analysis of the data for Live/Dead, percentage of live cells within the different scaffolds compared for ANOVA.

Treatment →	Collagen scaffolds fabricated using convention al method	Scaffolds faricated with the "magnetic particle string" method without magnetic field treatment	Scaffolds faricated with the "magneti c particle string" method	CMP functionalize d GMNP scaffolds without magnetic field treatment	Scaffolds Fabricate d with the "magneti c anchor" method	Pooled Total
observations N	2	2	2	2	2	10
sum ∑xi∑xi	156.4	154.9	153.5	176.5	170.2	811.5
mean ¯xx¯	78.2	77.45	76.75	88.25	85.1	81.15
sum of squares ∑x2i∑xi2	12,230.80	12,019.45	11,810.77	15,582.25	14,505.80	66,149.0 7
sample variance s2s2	0.32	22.445	29.645	6.125	21.78	32.8717
sample std. dev. ss	0.5657	4.7376	5.4447	2.4749	4.6669	5.7334

std. dev. of mean SE¯xSEx¯	0.4	3.35	3.85	1.75	3.3	1.8131

Table S8. The Tukey post hoc test of the ANOVA test performed for percentage of live cells within the different scaffolds compared

treatments	Tukey HSD	Tukey HSD	Tukey HSD
pair	Q statistic	*p*-value	inferfence
Collagen scaffolds fabricated using conventional method vs Scaffolds faricated with the "magnetic particle string" method without magnetic field treatment	0.2646	0.899995	insignificant
Collagen scaffolds fabricated using conventional method vs Scaffolds faricated with the "magnetic particle string" method	0.5116	0.899995	insignificant
Collagen scaffolds fabricated using conventional method vs CMP functionalized GMNP scaffolds without magnetic field treatment	3.5462	0.226144	insignificant
Collagen scaffolds fabricated using conventional method vs Scaffolds Fabricated with the "magnetic anchor" method	2.4347	0.496392	insignificant
Scaffolds faricated with the "magnetic particle string" method without magnetic field treatment vs Scaffolds faricated with the "magnetic particle string" method	0.247	0.899995	insignificant
Scaffolds faricated with the "magnetic particle string" method without magnetic field treatment vs CMP functionalized GMNP scaffolds without	3.8109	0.186019	insignificant

magnetic field treatment			
Scaffolds faricated with the "magnetic particle string" method without magnetic field treatment vs Scaffolds Fabricated with the "magnetic anchor" method	2.6994	0.417046	insignificant
Scaffolds faricated with the "magnetic particle string" method vs CMP functionalized GMNP scaffolds without magnetic field treatment	4.0579	0.155185	insignificant
Scaffolds faricated with the "magnetic particle string" method vs Scaffolds Fabricated with the "magnetic anchor" method	2.9464	0.350081	insignificant
CMP functionalized GMNP scaffolds without magnetic field treatment vs Scaffolds Fabricated with the "magnetic anchor" method	1.1115	0.899995	insignificant
